# A case of lethal suicidal intoxication with propafenone and diazepam

**DOI:** 10.1111/anec.13111

**Published:** 2024-03-04

**Authors:** Shuangbing Yan, Ting Xin, Xiaojie Luo, Yu Wang, Bingwei Chen

**Affiliations:** ^1^ Department of Cardiology Tianjin First Central Hospital Tianjin China; ^2^ Department of Emergency Tianjin First Central Hospital Tianjin China

**Keywords:** case report, diazepam, intoxication, myocardial non‐excitability, propafenone

## Abstract

Diazepam poisoning is a common emergency situation, but propafenone poisoning is relatively rare. We reported a case of propafenone poisoning combined with diazepam. An 18‐year‐old female patient was admitted to our hospital with an overdose of oral propafenone and diazepam. The patient was treated with medication that proved to be useful, but the sinus rhythm could not be recovered, and cardiac arrest occurred. A bipolar temporary pacemaker and extracorporeal membrane oxygenation (ECMO) were installed. However, even with multiple electrode positions, effective capture could not be achieved. The patient eventually died. We should be alert to the possibility of co‐poisoning.

## 1 INTRODUCTION

Propafenone is a class IC antiarrhythmic agent, which is a fast sodium channel blocker with weak β‐adrenoceptor‐blocking activity and calcium‐channel antagonist properties. Propafenone has been demonstrated effective for ventricular arrhythmia and supraventricular tachycardia (Ledda et al., 1981). Diazepam is a benzodiazepine drug, which is prescribed for anxiety, epilepsy, and muscle spasms (Calcaterra & Barrow, 2014). Diazepam poisoning is a common emergency situation, but propafenone poisoning is relatively rare. In this paper, we presented a case of propafenone poisoning combined with diazepam.

## 2 CASE REPORT

**FIGURE 1:**
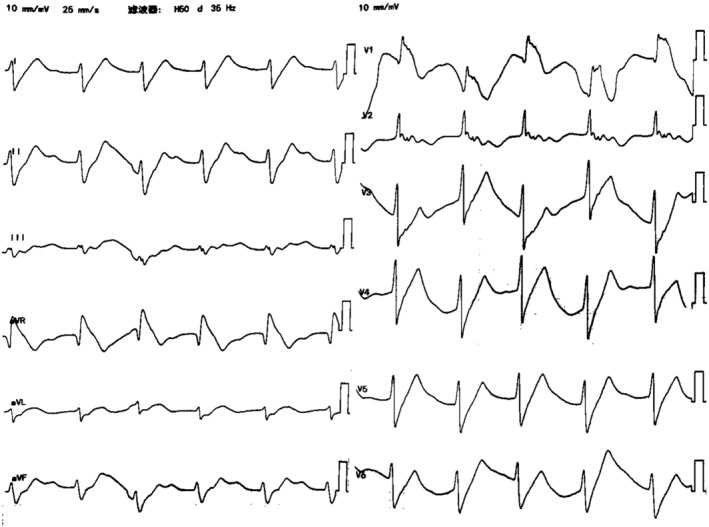
ECG at 2 h after ingestion of the drugs, showing P wave was not visible, widened QRS complexes, and prolonged QT interval.

An 18‐year‐old female patient, who had a history of depression and premature ventricular contractions with therapy including oral propafenone and diazepam respectively, was admitted to the local hospital, due to vomiting, by her family. On the way to the local hospital, the patient experienced convulsions and syncope fleetingly, and she regained consciousness 2 min later. Manual chest compressions and assisted ventilation with an oxygen face mask were administered in the ambulance. The patient took large doses of propafenone and diazepam (the exact dose was unknown) in an attempt to kill herself and arrived at the local hospital 2 h later. The electrocardiogram (ECG) of the local hospital showed that the P wave was not visible, widened QRS complexes, and prolonged QT interval (Figure 1). We considered that there might be two possibilities: ventricular escape rhythm or junctional rhythm with aberrant intraventricular conduction. On arrival at the local hospital, the patient suffered convulsions and syncope again. The ECG monitor was showing that the heart rate was 60 beats/min, the blood pressure was 70/40 mmHg, and pulse oximetry was 70%. Arterial blood gas analysis showed that the pH was 7.42, PaCO_2_ was 38.6 mmHg, PaO_2_ was 52.8 mmHg, oxygen saturation was 73.0%, BE was 2.1 mmol/L, K^+^ was 3.9 mmol/L, and ${{\mathrm{HCO}}_{3}}^{-}$ was 24.0 mmol/L. The patient underwent endotracheal intubation immediately. Dopamine and gastric lavage were then given. Echocardiography showed that both the atrial and ventricular diameters were within the normal range, while the left ventricular ejection fraction (LVEF) was as low as 36%. After partial clinical stabilization, the patient was transferred to the emergency room of our hospital by an ambulance.

**FIGURE 2:**
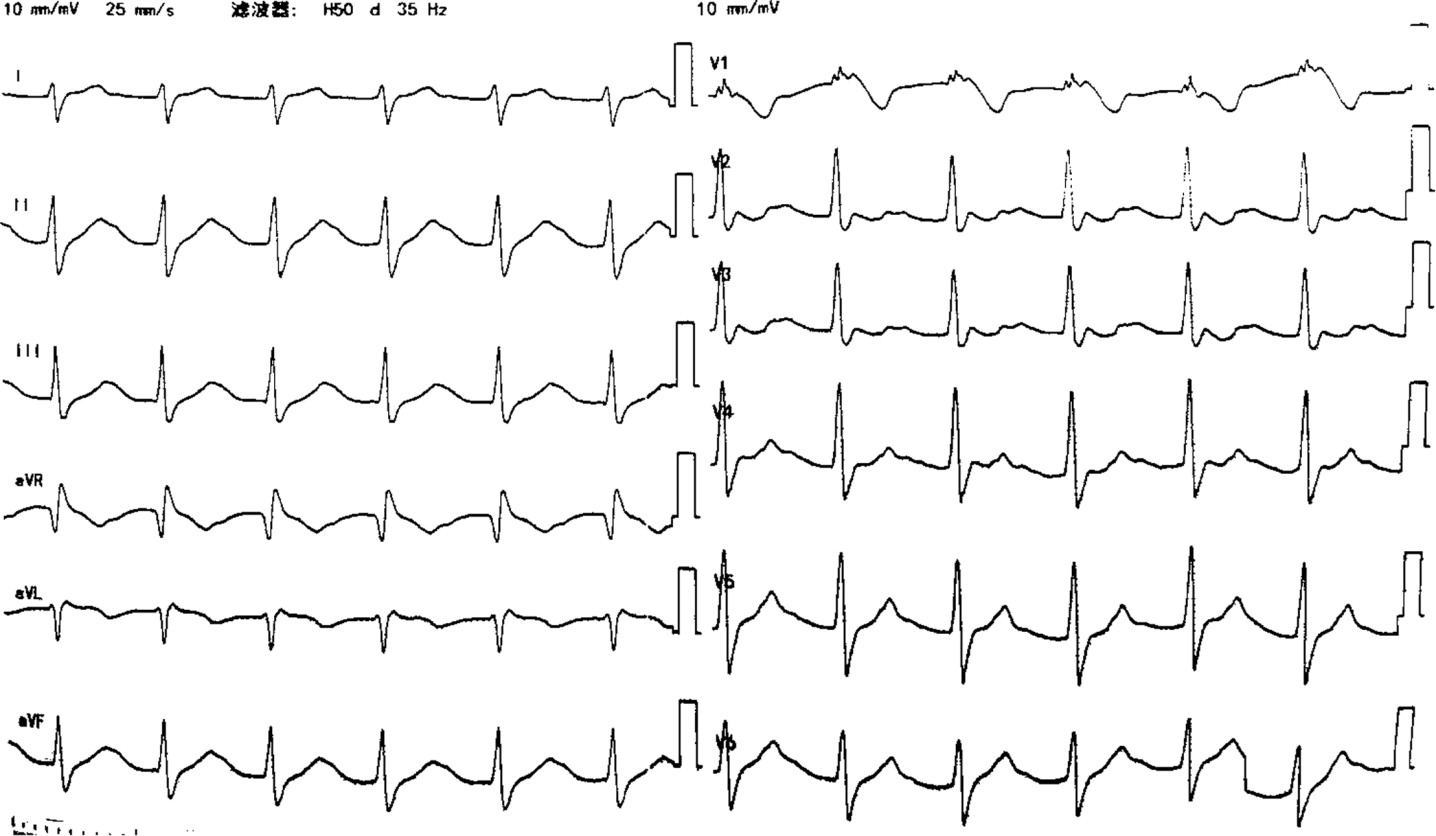
Four hours after ingesting the drugs, ECG showed a sinus rhythm with the heart rate of 75 beats/min, grade I atrioventricular block, and complete right bundle branch block.

**FIGURE 3:**
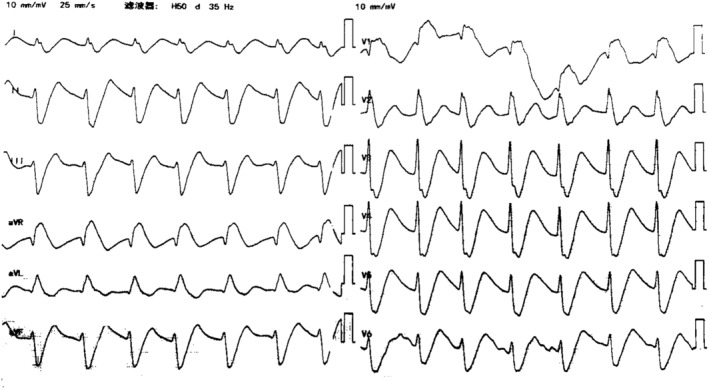
ECG of 9 h after ingesting the drugs showing that P wave was not visible, widened QRS complexes, and prolonged QT interval.

Four hours after ingesting the drugs, the patient, who was conscious of stable vital signs, arrived at our emergency room. Toxicology tests around 5 h after taking the drugs showed blood concentrations of 1.6 mg/L for propafenone and 1.1 mg/L for diazepam (the dose for treatment of propafenone was <1.0 mg/L, and the toxic dose of diazepam was >0.5 mg/L). ECG showed a sinus rhythm with the heart rate of 75 beats/min, grade I atrioventricular block, and complete right bundle branch block (Figure 2). Laboratory tests showed that liver function, renal function, and serum potassium were normal. Arterial blood gas analysis showed that the pH was 7.35 (normal range: 7.35–7.45), PaCO_2_ was 38.70 mmHg (normal range: 35–45 mmHg), PaO_2_ was 405 mmHg (normal range: 80–100 mmHg), K^+^ was 4.10 mmol/L (normal range: 3.5–4.5 mmol/L), and ${{\mathrm{HCO}}_{3}}^{-}$ was 22.30 mmol/L (normal range: 21–26 mmol/L). The patient was given gastric lavage again. Nine hours after ingesting the drugs, the patient suddenly experienced convulsions and syncope. The ECG monitor was showing that the heart rate was 54 beats/min, and the blood pressure dropped to 50/30 mmHg. Similar symptoms occurred twice. Arterial blood gas analysis showed marked metabolic acidosis (pH: 7.19, PaCO_2_: 25.6 mmHg, PaO_2_: 254.8 mmHg, lactic acid: 7.10 mmol/L [normal range: 1.0–1.7 mmol/L], BE: −12.70 mmol/L [normal range: −3 to +3 mmol/L], and K^+^: 3.2 mmol/L, ${{\mathrm{HCO}}_{3}}^{-}$: 15.10 mmol/L). The patient was given epinephrine, norepinephrine, sodium bicarbonate, dopamine, rehydration, and continuous mechanical ventilation. ECG showed that the P wave was not visible; widened QRS complexes and prolonged QT interval after the above drugs were used (Figure 3). We considered that there might be two possibilities. It might be a ventricular escape rhythm with a heart rate of 85 beats per minute, simply because the patient was given drugs to raise the heart rate. Otherwise, it might be a junctional rhythm with an aberrant intraventricular conduction, and the intraventricular conduction block was more severe than before. The ECG monitor showed a rapid drop in heart rate to 40 beats per minute and even cardiac arrest. Immediate chest compressions were performed. A bipolar temporary pacemaker was implanted urgently and extracorporeal membrane oxygenation (ECMO) was performed. We did not test the atrial threshold, and ventricular capture was never available. Even with multiple electrode positions and medicine such as epinephrine, atropine, and isoproterenol, effective capture could not be achieved.

The patient was taken to the intensive care unit after the operation. Hemoperfusion (HP) and continuous venovenous hemofiltration (CVVH) were started subsequently. Norepinephrine, dopamine, high‐dose insulin and glucose, sodium bicarbonate, and rehydration were given. Echocardiogram revealed an incongruous left ventricular motion, a reduced left ventricular systolic function, and a 31% LVEF. The last arterial blood gas analysis showed respiratory alkalosis with metabolic acidosis (pH: 7.47, PaCO_2_: 24.80 mmHg, PaO_2_: 317 mmHg, lactic acid: 5.50 mmol/L, BE: −5.33 mmol/L, K^+^: 3.61 mmol/L, and ${{\mathrm{HCO}}_{3}}^{-}$: 17.50 mmol/L). The patient had been on ECMO support (ECMO support was maintained for around 11 h) and continuous mechanical ventilation. But unfortunately, the patient remained in a deep coma with hemodynamic instability. The heart rate remained very slow with repeated long pauses, and effective pacing was never achieved. Even with a system of medical care insurance, the patient's family still needed to bear exorbitant costs for ECMO therapy, which was far beyond their financial means. It was a great pity that her family finally gave up the rescue. The patient eventually died 20 h after ingesting the drugs.

## 3 DISCUSSION

Propafenone blocks the fast inward sodium current during phase 0 of the action potential, which slows down the conduction of atrial, ventricular, and Purkinje fibers, and prolongs the duration of QRS complex. Propafenone also has negative inotropic activity at high concentrations (Ledda et al., 1981). Arrhythmias include bradycardia, ventricular tachycardia, ventricular flutter, ventricular fibrillation, and cardiac arrest (Wozakowska‐Kaplon & Stepien‐Walek, 2010). The pharmacokinetic properties of propafenone have shown the marked interindividual variability in extensive and poor metabolizers. Peak plasma concentration occurs at 2–3 h after oral delivery (Harron & Brogden, 1987). Diazepam is a bioavailable, widely distributed, and lipid‐soluble central nervous system penetrant compound. Peak plasma levels are reached after 30–90 min via oral delivery (Calcaterra & Barrow, 2014). The manifestation of diazepam overdose is inhibition of the central nervous system, from lethargy to coma (Bellantuono et al., 2013). Diazepam poisoning is a common emergency situation, but propafenone poisoning is relatively rare. There have been no reports of compound poisoning of propafenone and diazepam. Toxicology results showed significant excess plasma concentrations of propafenone and diazepam in this case, confirming the dual drug poisoning.

The significant feature of this case was that the effective pacing could not be obtained. The main reason was that propafenone significantly inhibited the automaticity of cardiomyocytes and Purkinje fibers, leading to an increase in the threshold for pacemaker capture. In addition, both diazepam and propafenone inhibited respiration, which could aggravate metabolic acidosis and cause potassium outflow. Severe hyperkalemia could deteriorate conduction block and cause malignant arrhythmias. Therefore, we speculated that the poor perception of temporary pacemaker was associated not only with high overdose of propafenone, but also with diazepam intoxication. After treatment, the patient appeared to improve briefly and the ECG showed a narrow QRS complex, but it was an illusion. The patient appeared to deteriorate soon and the abnormal wide QRS complex appeared again. We suspected there were three reasons. Firstly, the stomach contents were pushed into the intestine because of excessive gastric lavage, leading to a large absorption of propafenone in the small intestine. The patient might suffer from secondary poisoning. Secondly, as soon as the patient arrived in our emergency room, we performed HP and CVVH. But unfortunately, it was 4 h after taking the medicine. Propafenone was not cleared from the blood as soon as possible. Thirdly, the patient had been in a state of hypotension, and the possibility of latent acidosis could not be ruled out. The combined diazepam poisoning increased the difficulty of rescue. Diazepam was also described to have drug interactions with beta‐adrenoceptor antagonists (Aronson, 2016), and this might be the cause of the aggravated irreversible condition of the patient. The cause of the sudden cardiac arrest was the significant inhibition of excitability and conductivity of cardiomyocytes caused by propafenone overdose, which was the ultimate cause of death.

There is still no specific antidote for propafenone poisoning. Early gastric lavage and activated charcoal can remove drugs from the intestines and reduce absorption. Cardiopulmonary resuscitation is essential to ensure the blood supply of vital organs. Rational use of positive inotropic drugs and vasoactive agents such as adrenaline, norepinephrine, and isoproterenol are also necessary. For symptomatic bradycardia, a temporary pacemaker has also been shown to be effective. Case reports showed that high‐dose insulin and glucose, intravenous lipid emulsion, glucagon, sodium bicarbonate, and calcium gluconate were effective in propafenone poisoning (Bayram et al., 2013, 2015; Chen & Yang, 2017; Ovaska et al., 2010). Our center tried not only gastric lavage, manual chest compressions, vasoactive agents, sodium bicarbonate, high‐dose insulin and glucose, a bipolar temporary pacemaker, ECMO support, and continuous mechanical ventilation, but also HP combined with CVVH, which had been proven to be effective by other centers (Ling et al., 2018).

Double poisoning led to the complexity and confidentiality of the disease. The patient got better and then got worse again, which made us unable to make accurate judgments. Although we tried almost everything that proved to be useful, the death was unavoidable. This was the first reported case of combined poisoning of propafenone and diazepam. We should be alert to the possibility of co‐poisoning when we find similar situations in arrhythmia patients. And it should be recommended that these patients must be closely and vigilantly monitored even after a seeming recovery.

## AUTHOR CONTRIBUTIONS

Shuangbing Yan, Bingwei Chen, and Yu Wang participated in the design of the study. Shuangbing Yan, Ting Xin, and Xiaojie Luo participated in data collection. Shuangbing Yan, Bingwei Chen, and Xiaojie Luo participated in the literary search. Shuangbing Yan and Bingwei Chen wrote the manuscript. All authors were involved in the preparation of this manuscript and have read and agreed to the published version of the manuscript.

## FUNDING INFORMATION

This work was supported in part by the Tianjin Key Medical Discipline (especially) Construction Project (TJYXZDXK‐054B). The funding bodies played no role in the design of the study, collection, analysis, and interpretation of the data.

## CONFLICT OF INTEREST STATEMENT

The authors declare that they have no competing interests.

## ETHICS STATEMENT

This study was approved by the Institutional Review Board at Tianjin First Central Hospital. And the procedures were conducted according to the principles of the Helsinki Declaration.

## INFORMED CONSENT

Written informed consent was obtained from the patient's family for the publication of this case report and any accompanying figure.

## Data Availability

All data generated or analyzed during this study are included in this article. Further requirements can be directed to the corresponding authors.
